# Prognostic analysis of patients with gastric cancer based on N^6^-methyladenosine modification patterns and tumor microenvironment characterization

**DOI:** 10.3389/fphar.2024.1445321

**Published:** 2024-08-09

**Authors:** Miaomiao Huo, Min Zhang, Jingyao Zhang, Yong Wang, Ting Hu, Tianyu Ma, Yinuo Wang, Baowen Yuan, Hao Qin, Xu Teng, Hefen Yu, Wei Huang, Yan Wang

**Affiliations:** ^1^ Key Laboratory of Cancer and Microbiome, State Key Laboratory of Molecular Oncology, National Cancer Center/National Clinical Research Center for Cancer/Cancer Hospital, Chinese Academy of Medical Sciences and Peking Union Medical College, Beijing, China; ^2^ Department of Ultrasound, National Cancer Center/National Clinical Research Center for Cancer/Cancer Hospital, Chinese Academy of Medical Sciences and Peking Union Medical College, Beijing, China; ^3^ Beijing Key Laboratory of Cancer Invasion and Metastasis Research, Department of Biochemistry and Molecular Biology, School of Basic Medical Sciences, Capital Medical University, Beijing, China

**Keywords:** STAD, m^6^A modification, TME infiltration, WGCNA, LASSO

## Abstract

**Background:**

Cancers arise from genetic and epigenetic abnormalities that affect oncogenes and tumor suppressor genes, compounded by gene mutations. The N6-methyladenosine (m^6^A) RNA modification, regulated by methylation regulators, has been implicated in tumor proliferation, differentiation, tumorigenesis, invasion, and metastasis. However, the role of m^6^A modification patterns in the tumor microenvironment of gastric cancer (GC) remains poorly understood.

**Materials and methods:**

In this study, we analyzed m^6^A modification patterns in 267 GC samples utilizing 31 m^6^A regulators. Using consensus clustering, we identified two unique subgroups of GC. Patients with GC were segregated into high- and low-infiltration cohorts to evaluate the infiltration proportions of the five prognostically significant immune cell types. Leveraging the differential genes in GC, we identified a “green” module via Weighted Gene Co-expression Network Analysis. A risk prediction model was established using the LASSO regression method.

**Results:**

The “green” module was connected to both the m^6^A RNA methylation cluster and immune infiltration patterns. Based on “Module Membership” and “Gene Significance”, 37 hub genes were identified, and a risk prediction model incorporating nine hub genes was established. Furthermore, methylated RNA immunoprecipitation and RNA Immunoprecipitation assays revealed that YTHDF1 elevated the expression of DNMT3B, which synergistically promoted the initiation and development of GC. We elucidated the molecular mechanism underlying the regulation of DNMT3B by YTHDF1 and explored the crosstalk between m^6^A and 5mC modification.

**Conclusion:**

m^6^A RNA methylation regulators are instrumental in malignant progression and the dynamics of tumor microenvironment infiltration of GC. Assessing m^6^A modification patterns and tumor microenvironment infiltration characteristics in patients with GC holds promise as a valuable prognostic biomarker.

## 1 Introduction

RNA methylation plays a significantly role in normal cellular homeostasis and pathological conditions (Han et al., 2020), in which N6-methyladenosine (m^6^A) is the most prevalent in eukaryotic cells and has gained increasing attention because of its presence in mRNA, lncRNAs, and miRNA (Dominissini et al., 2012; Meyer et al., 2012; Ontiveros et al., 2019; Huang et al., 2020). Recently, m^6^A modification, a dynamic and reversible epigenetic process, has attracted considerable attention. This modification is orchestrated by regulators commonly referred to as “writers”, “erasers”, and “readers”. The methylation process is specifically catalyzed by methyltransferases, or “writers”, which encompass enzymes such as METTL3, METTL14, WTAP, and METTL16. Conversely, the demethylation process is executed by demethylases such as FTO and ALKBH5, termed “erasers”. Additionally, there is a set of RNA-binding proteins encompassing, but not restricted to, YTHDC1, YTHDC2, YTHDF1, YTHDF2, YTHDF3, IGF2BP1 (Zhang et al., 2020). Numerous studies have reported that m^6^A modifications are common in cancer. Such modifications profoundly influence tumorigenesis and tumor progression by disrupting cellular pathways; promoting cell proliferation, self-renewal, and tumor metastasis; and leading to aberrations in immunomodulation (Pinello et al., 2018; Tong et al., 2018; Chen et al., 2020; Dong and Cui, 2020; Haruehanroengra et al., 2020). Furthermore, programmed cell death pathways, which are closely associated with the cancer initiation, progression, and resistance, have highly complex links to m^6^A modification (Liu et al., 2022).

Gastric cancer (GC) is one of the most prevalent digestive tract cancers globally, with an incidence rate of 5.6% and a mortality rate of 7.7%, ranks the top five in both categories (Sung et al., 2021). With progress in biological information technology and medical means, genome analysis has become the main method for identifying new biological targets in GC (Cancer Genome Atlas Research, 2014; Oh et al., 2018). The overall abundance of m^6^A mRNA in human GC tissues is significantly higher compared to normal tissue (Wang Q. et al., 2020). Studies have found that tumor progression is not solely attributed to genetic and epigenetic modifications in tumor cells. The tumor microenvironment (TME), on which cancer cells rely for growth and survival, plays a pivotal role. Cancer cells modify various biological behaviors through direct and indirect interactions. As our comprehension of the complexities and variations within TME deepening, increasing evidence highlights the pivotal role in tumor progression and immune evasion, as well as its influence on responses to immunotherapy (Quail and Joyce, 2013; Ali et al., 2016). Consequently, there is a growing emphasis on studying biomarkers that can predict responses to immune checkpoint blockade therapies, aiming to enhance precision immunotherapy strategies. Virtually, m^6^A modification alterations intrinsically affect immune cells and extrinsically affect immune cell responses in the TME (Li et al., 2022). m^6^A is closely related to macrophage phenotype and dysfunction (Zhu X. et al., 2023). In GC, m^6^A modification plays a non-negligible role in characterizing TME infiltration, both in terms of diversity and complexity (Zhang et al., 2020).

In this study patient samples are divided into two subgroups by conducting a consistent cluster analysis of the expression profiles of 31 m^6^A RNA methylation factors from patients with GC found in the TCGA database. Based on the proportion of infiltration from the five immune cell types related to prognosis, patients with GC were categorized into high-infiltration and low-infiltration groups, where there was a significant difference in prognosis. Using WGCNA analysis of the differential genes in GC, we identified a module associated with both the m^6^A methylation cluster and the immune infiltration classification. Hub genes were isolated from this module using module membership (MM) and gene significance (GS). By leveraging these hub genes, we employed LASSO regression to develop a risk prediction model. Based on the risk score of this model, we further categorized the samples into high-risk and low-risk groups. Furthermore, we validated the isolated hub genes and found that the m^6^A reader, YTHDF1, elevated the expression of DNMT3B, synergistically promoting the initiation and development of GC.

## 2 Materials and methods

### 2.1 GC datasets source and preprocessing

**FIGURE 1:**
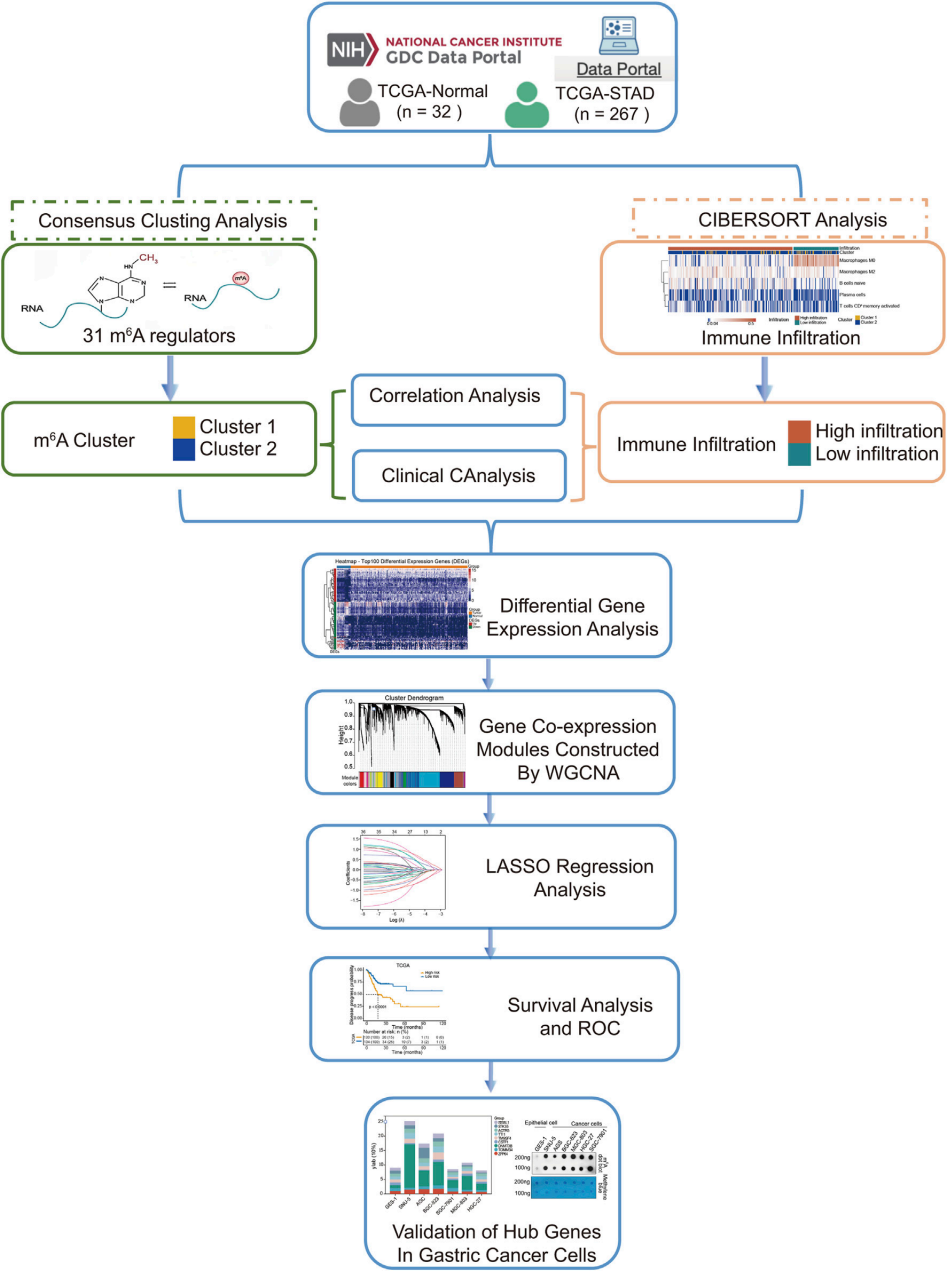
Flow chart of analysis.

We extracted gene expression datasets and associated clinical annotations from two renowned repositories: UCSC Xena (accessible at https://xenabrowser.net/datapages/) and cBioportal (available at http://www.cbioportal.org/). We included a cohort of 267 gastric cancer patient samples along with 32 samples derived from healthy individuals. These samples were repleted with pertinent survival data and detailed tumor staging, all sourced from The Cancer Genome Atlas-Stomach Adenocarcinoma (TCGA-STAD) database download from UCSC Xena. A comprehensive list of these details is provided in Table 1. We also integrated the GC dataset GSE62254 from the Gene Expression Omnibus, constituting an additional 300 GC patient samples; patients without survival information were excluded from further evaluation. The comprehensive sample data are presented in Table 2. The workflow of data analysis is shown in Figure 1.

**TABLE 1:** Clinical characteristics of the patients from TCGA-STAD.

Characteristics	Subtype	Case, n	Ratio, n (%)
Total		267	100
Gender	Female	94	35.20
	Male	173	54.80
Stage	I	42	15.73
	II	94	35.21
	III	108	40.45
	IV	23	8.61
Grade	G1	7	2.62
	G2	97	36.33
	G3	158	59.18
	G4	5	1.87
Age	≥60	169	63.30
	<60	95	35.58
	NA	3	1.12

**TABLE 2:** Clinical characteristics of the patients from GSE62254.

Characteristics	Subtype	Case, n	Ratio, n (%)
Total		300	100
Age	≥60	194	64.67
	<60	106	35.33
Gender	Male	199	63.33
	Female	101	33.67
Stage	I	30	10
	II	97	32.33
	III	96	32.00
	IV	77	25.67

### 2.2 Consistent cluster analysis

A list of the 31 m^6^A regulators is provided in the Supplementary Material (Table S1). Utilizing the R package “ConsensusClusterPlus” (Wilkerson and Hayes, 2010), we performed a consistent clustering analysis with a robust setting of 100 iterations and an 80% resampling rate, leveraging the Pearson correlation as the chosen distance metric. This rigorous analysis stratified the samples into two distinct clusters: Cluster 1 and Cluster 2.

A Principal Component Analysis (PCA) was performed to visually discern and compare the underlying variations between the two clusters. This dimensionality reduction technique allowed us to capture the essence of variance in the data and offered a clearer perspective on the differences in expression patterns. The Wilcoxon test was used to ascertain the specific m^6^A RNA methylation regulators that exhibited significant differential expression between Cluster 1 and Cluster 2. This non-parametric statistical test, tailored for datasets that did not necessarily follow a normal distribution, enabled us to rigorously compare the expression levels of each regulator between the two defined clusters.

### 2.3 Survival analysis of m^6^A cluster and subgroup functional pathway analysis

Survival analysis encompasses statistical methods dedicated to exploring the expected time until one or more events occur. We used the Kaplan-Meier method to generate survival curves, and the log-rank test was used to identify prognostic factors correlated with survival, with significance determined at a threshold of *p* < 0.05. Furthermore, for enrichment analysis, we leveraged the “GSVA” package in R, which employs an unsupervised, non-parametric approach (Hanzelmann et al., 2013; Shen et al., 2022). Significance level of *p* < 0.05 was considered statistically significance.

### 2.4 Estimation of the proportion of TME cell infiltration in GC

CIBERSORT (accessible at https://cibersort.stanford.edu/) was synergistically paired with the LM22 signature matrix, facilitating the estimation of the proportion of human hematopoietic cell phenotypes within the 22 samples categorized from both the high-risk and low-risk patient cohorts. Notably, the cumulative proportion of all the inferred immune cell types within each sample was 1. Subsequently, to identify the immune cells with significant prognostic implications, the proportions of these various immune cells were subjected to univariate Cox regression analyses.

### 2.5 Identification of differentially expressed genes (DEGs)

Differentially expressed genes between 267 tumor samples and 32 normal samples were identified using the Limma R package (Smyth, 2004). Genes were considered differentially expressed based on thresholds of |log_2_FC| > 0.585 and FDR < 0.01.

### 2.6 Weighted gene co-expression network analysis (WGCNA)

WGCNA is an advanced bioinformatics approach aimed at deciphering the complex patterns of gene expression data (Yin et al., 2020). By calculating the correlation between the module eigengenes and the clinical traits of interest, biologically relevant modules were identified. Within the modules significantly associated with clinical traits, hub genes were identified based on their connectivity, highlighting those with potential key roles in the module’s biological function.

### 2.7 Construction and validation of the prognosis model

For the identified hub genes, the LASSO method was employed to select prognostically relevant genes and construct a prognostic model. The systematically derived risk-scoring formula was as follows:

$$\text{RiskScore }\left(\text{RS}\right)=\text{EXPZFP}64*\left(-0.044\right)+\text{EXPTOMM}34*\left(-0.041\right)+\text{EXPDNMT}3B\;*\;\left(0.131\right)+\text{EXPCSTF}1*\;\left(-0.273\right)+\text{EXPTM}9\text{SF}4*\;\left(0.264\right)+\text{EXPTTI}1\;*\;\left(0.136\right)+\text{EXPACTR}5*\;\left(-0.255\right)+\text{EXPSTK}35*\;\left(-0.006\right)+\text{EXPSS}18L1*\;\left(-0.047\right)$$

Based on the median risk score derived from the model or the optimal cutoff value calculated using the surv_cutpoint function, patient samples were stratified into high-risk and low-risk groups. Kaplan-Meier survival analysis was used to assess the predictive capability of the model.

### 2.8 Cell culture and transfection

Cell lines AGS, BGC-823 and HGC-27 were obtained from Shanghai Institute of Biochemical Cell Science, Chinese Academy of Sciences. BGC-823 cells were cultured in Dulbecco’s modified Eagle’s medium (DMEM) and AGS cells were cultured in DMEM/F12. All the culture mediums were supplemented with 10% fetal bovine serum (FBS), 100 units/mL penicillin, and 100 mg/mL streptomycin (Gibco, 15140-122, United States). The cells were cultured in a constant temperature incubator equilibrated with 5% CO_2_ at 37°C. The sources and culture conditions of other cell lines are detailed in Supplementary Table S2. All experiments were performed with mycoplasma-free cells.

All plasmids were verified by DNA sequencing and transfected using TurboFect™ reagent (ThermoFisher, R0531, USA). SiRNAs for gene knockdown were transfected using Lipofectamine RNAiMAX reagent (Invitrogen, 13778-150, United States). The siRNA sequences used are listed in the Supplementary Table S3.

### 2.9 Real-time quantitative polymerase chain reaction (real-time qPCR) analysis

Total RNA was extracted using TRIzol reagent. Subsequently, reverse transcription was performed using the PrimeScript™ RT Master Mix (Takara, RR036A, Japan), according to the manufacturer’s instructions. qPCR was conducted using FastStart Universal SYBR Green Master (Roche, 4913914001, United States), where β-actin was analyzed as the loading control. The relative expression of target genes was calculated using the 2^−ΔΔCT^ method. The primers used are listed in Supplementary Table S4.

### 2.10 Western blot

Total proteins were separated via SDS-PAGE. The proteins were transferred onto the PVDF membrane and blocked using 5% skim milk at room temperature for 1 h, followed by immunoblotting with the indicated antibodies overnight, including anti-YTHDF1 (Proteintech, Cat No.17479-1-AP, China) and anti-DNMT3B (Cell Signaling Technology, 57868S, United States). After incubation with secondary antibodies for 1 h at room temperature, the membranes were washed and transferred onto an X-ray radiographic cassette and treated with ECL Super Signal™ West Pico PLUS (ThermoFisher, 34580, USA). Subsequently, the membranes were blotted onto X-ray films for visualization.

### 2.11 Methylated RNA immunoprecipitation (MeRIP) and RIP

The MeRIP-qPCR assay was conducted using a MeRIP assay kit, according to the manufacturer’s instructions (Bersinbio, China). The RIP assay was performed using approximately 2 × 10^7^ cells per sample, and the specific experimental steps were based on previously methods reported (Gagliardi and Matarazzo, 2016). The m^6^A sites of DNMT3B were predicted using SRAMP (http://www.cuilab.cn/sramp) (Zhou et al., 2016), and primers containing m^6^A sites were subsequently designed. The primers used are listed in Supplementary Table S5.

### 2.12 EdU assay

GC cells with overexpression or depletion of the indicated genes and control were seeded into 96-well plates at a density of 6 × 10^4^ cells per well. DNA proliferation was detected after the cells were cultured overnight using an EdU assay kit (RiboBio, Bes5203-1, China). Images were acquired using an inverted fluorescence microscope and statistically analyzed using ImageJ software.

### 2.13 Cell migration and invasion assay

For the cell migration assay, Transwell chamber filters were placed in 24-well plates. Cells transfected with indicated siRNAs were suspended in serum-free medium, and 8 × 10^4^ cells were seeded into the upper chamber of the wall, whereas the lower chamber was cultured in medium containing 15% FBS. Following incubation for 24 h, the cells were fixed with 4% paraformaldehyde for 20 min, then stained with 0.1% crystal violet after which the cells in the upper chamber were removed. Images were acquired under an inverted microscope and statistically analyzed using ImageJ software. For the cell invasion assay, Transwell chamber filters wrapped in 10% Matrigel (Corning, 354234, United States) were used.

### 2.14 Statistical analysis

Data analyses were performed using GraphPad Prism (version 9.1.1) and results are displayed as the mean ± SD. Student’s t-test was used to compare differences between the two groups. Potential m^6^A modification sites were predicted using SRAMP (http://www.cuilab.cn/sramp). Survival curves were plotted using the Kaplan-Meier “survival” package in R (version 3.4.3), where the log-rank test was used to assess statistical significance. Statistical significance was set at *p* < 0.05.

## 3 Results

### 3.1 Landscape of TME in GC and infiltration characteristics in distinct m^6^A modification patterns

**FIGURE 2:**
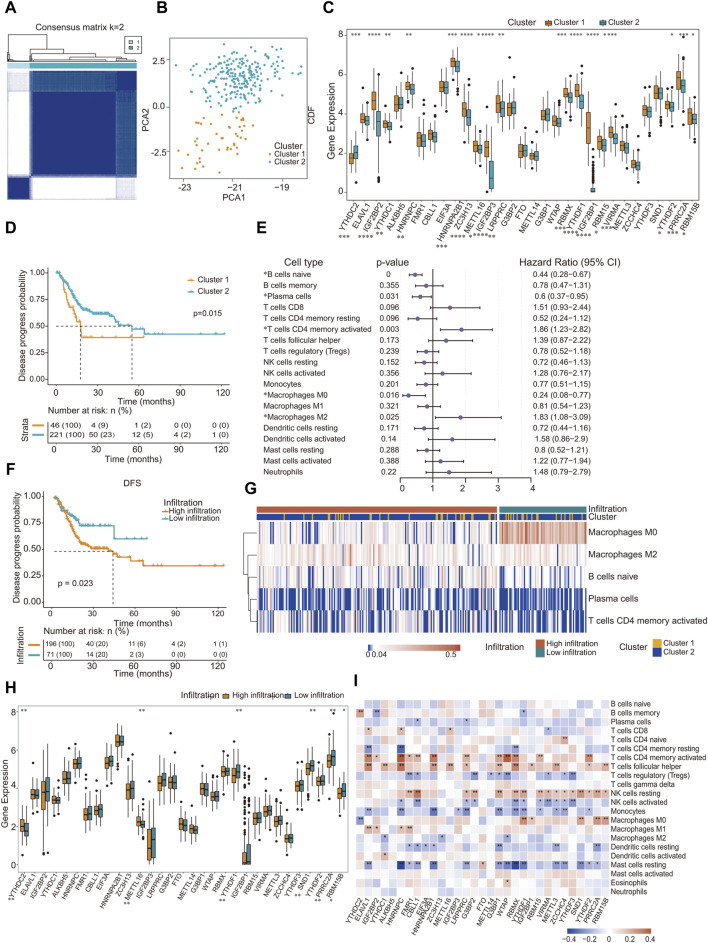
Landscape of TME in gastric cancer and infiltration characteristics in distinct m^6^A modification patterns. **(A)** All gastric cancer samples were divided into two groups (K = 2); **(B)** Under PCA algorithm, the two subgroups cluster 1 and cluster 2 showed significant difference; **(C)** The expression of 31 m^6^A regulators between 2 m^6^A regulators genes cluster: cluster 1 and cluster 2. Cluster 1, Orange; Cluster 2, blue. The upper and lower ends of the boxes represented inter quartile range of values. The lines in the boxes represented median value, and black dots showed outliers; **(D)** Kaplan-Meier curves for disease progress probability gastric cancer patients from two clusters; **(E)** Subgroup analysis estimating clinical prognostic value in different types of immune cell infiltration. The length of the horizontal line represents the 95% confidence interval for each group; **(F)** Kaplan-Meier curves for patients with high and low cohort. Log-rank test shows an overall *p* = 0.023; **(G)** Heat map of distribution of five kinds of immune cells in different immune invasion groups; **(H)** Gene expression level of m^6^A methylation regulator in different immune infiltration groups; **(I)** Correlation between the expression of m^6^A regulator and the proportion of immune cells. ∗*p* < 0.05, ∗∗*p* < 0.01, ∗∗∗*p* < 0.001; two-tailed unpaired *t*-test.

m^6^A methylation modification patterns mediated by 31 regulators were analyzed in patient-derived GC samples. The m^6^A methylation mediated by regulators classified as “writers”, “erasers” and “readers” is a dynamic reversible process (Supplementary Figure S1A). We employed the R package “ConsensusClusterPlus”, a tool specifically designed for the robust class discovery and visualization of gene expression datasets, and all GC samples into two distinct groups (Figure 2A). PCA was used to evaluate these groups, revealing significant differences between them (Figure 2B). We utilized the PAM clustering method, with the sample correlation coefficient calculated using Pearson correlation. As depicted in the cumulative consistency distribution map (Supplementary Figures S1B, S1C), there was a noticeable increase in the broken line beyond K = 2, prompting us to categorize all GC samples into two distinct groups. We conducted a Wilcoxon test on the expression levels of 31 m^6^A regulators across both groups. The findings highlighted that 17 methylation factors, including YTHDC2, IGF2BP2, YTHDC1, and HNRNPC, exhibited significant variance in expression between two sample groups (Figure 2C).

Survival outcomes were assessed using the Kaplan-Meier log-rank test. Factors defined as *p* < 0.05 were determined to be prognostic determinants pertinent to survival rates. Incorporating the survival data of the patients, a pronounced disparity in prognosis was observed between the two sample clusters: cluster 1 (N = 46) and cluster 2 (N = 221) (Figure 2D). Interestingly, no significant variance in mutational count was observed between these clusters (Supplementary Figure S1D). Pathway enrichment analysis was performed on distinct subgroups using the GSVA package in R, with significance adjusted to *p* < 0.05. The findings revealed associations of distinct subgroups with several pathways and functional modalities, including the p53 pathway, interferon γ response, and reactive oxygen species pathways (Table 3; Supplementary Figure S1E).

**TABLE 3:** Pathway enrichment analysis of distinct subgroups.

Pathways	Log_2_FC	*p*-value
p53 pathway	0.228439016	0.000840047
Interferon Gama response pathway	0.270472645	0.001019054
Reactive oxygen species pathway	0.24754051	0.001019054
IL6/JAK/STAT3 signaling pathway	0.223662681	0.004037033
Allograft rejection pathway	0.234698376	0.004037033
Inflammatory response pathway	0.198208664	0.010601234
Interferon alpha response pathway	0.22826432	0.012046511

Using the CIBERSORT algorithm in tandem with the LM22 signature matrix, we ascertained the proportional abundance of 22 immune cell subtypes within GC specimens. Univariate Cox regression analysis was performed on 19 immune cell categories. Three immune cell types, namely CD4 naïve (detected in only three samples), T cell gamma delta (present in 18 samples), and eosinophils (identified in 21 samples), were excluded from the analysis because of their infrequent occurrence, rendering them unsuitable for a statistically robust Cox regression analysis. Our investigation revealed that naïve B cells, plasma cells, activated CD4^+^ memory T cells, and five additional immune cell types exhibited a pronounced correlation with prognosis (Figure 2E). By incorporating patient survival information, we observed a pronounced difference in prognosis between the two sample types characterized by distinct immune infiltration (Figure 2F). Subsequently, based on the infiltration metrics of these five salient immune cells, patient specimens were stratified into two discrete clusters, termed high-infiltration and low-infiltration, using the K-means unsupervised clustering technique. Within these classifications, the sample distribution was 196 with high infiltration and 71 with low infiltration (Figure 2G).

According to the immune infiltration grouping, Wilcoxon test was performed on the expression of 31 m^6^A regulators in the two groups of samples. Our analysis revealed that the expression of m^6^A methylation factors, notably YTHDC2, METTL16, and YTHDF1, was significantly different between the two groups (Figure 2H). Subsequently, the Pearson correlation coefficient between the 31 m^6^A RNA methylation factors and the infiltration proportion of the 22 immune cell types was calculated using the R package psych. Remarkably, most m^6^A methylation factors were correlated with the infiltration proportions of certain immune cells, particularly CD4 memory-activated T cells and follicular helper T cells (Figure 2I).

### 3.2 Correlation of hub genes with m^6^A regulators and immune infiltration

**FIGURE 3:**
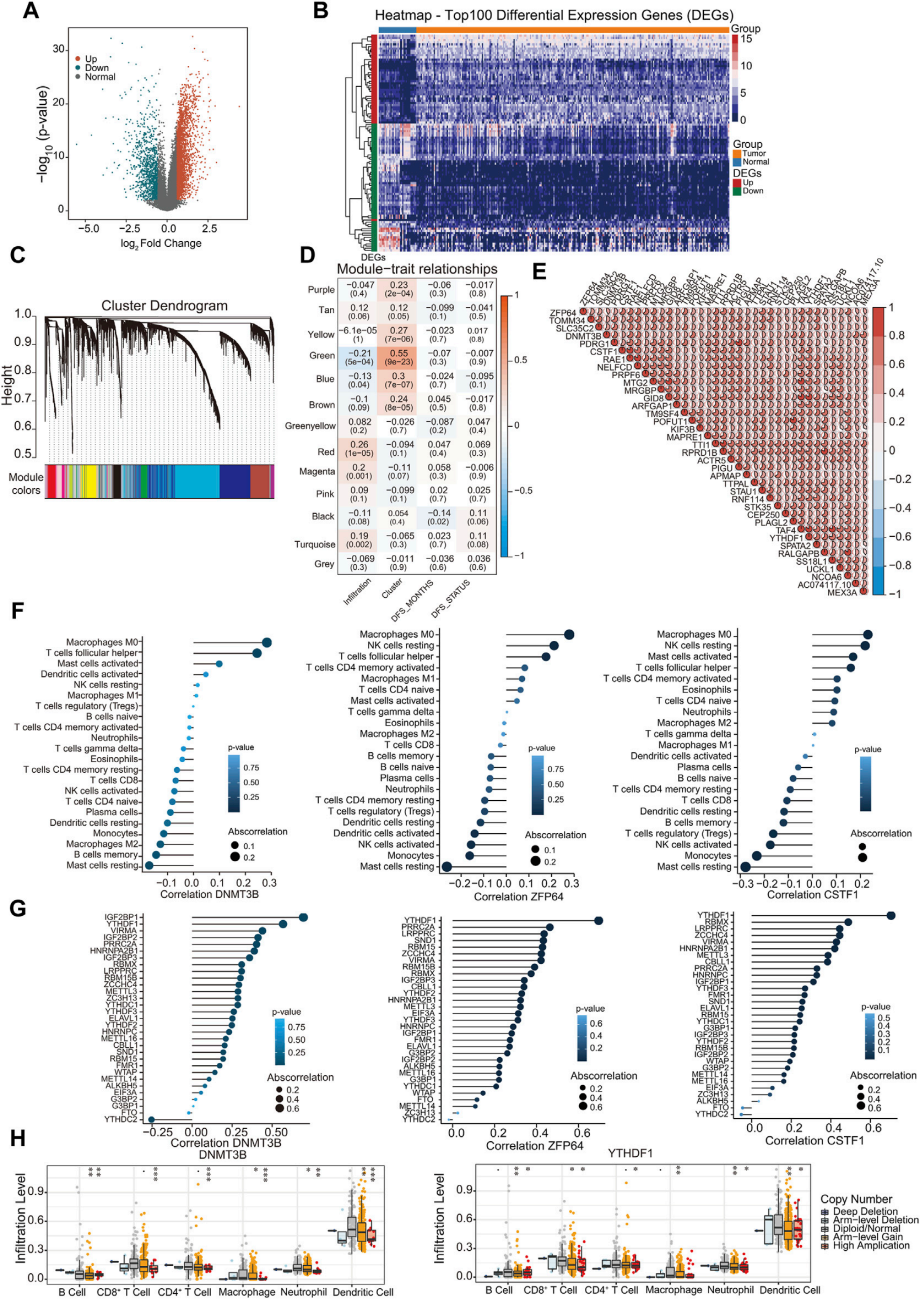
Correlation of hub genes with m^6^A regulators and immune infiltration. **(A)** Display of differential gene volcano map; **(B)** The heat map shows the top 100 |log_2_FC| of differential genes; **(C)** Gene dendrogram and module colors; **(D)** Module-trait relationship; **(E)** Correlations between hub genes. Negative correlation was marked with blue and positive correlation with red; **(F)** The correlation between immune infiltration types and hub genes DNMT3B, ZNF64, CSTF1; **(G)** The correlation between m^6^A regulators and hub genes DNMT3B, ZNF64, CSTF1; **(H)** Analysis of the differences in immune infiltration and SCNAs among hub genes. ∗*p* < 0.05, ∗∗*p* < 0.01, ∗∗∗*p* < 0.001; two-tailed unpaired *t*-test.

The gene expression profile data of 267 patient samples and 32 control samples from the TCGA database were used for differential gene screening and differential gene expression analysis. According to the multiple differences (|log_2_FC| > 0.585) and significance threshold (FDR < 0.01), 4,391 DEGs were screened using the R package Lima, including 3,232 upregulated and 1,159 downregulated genes (Figures 3A, B).

Leveraging 4,391 DEGs, we constructed a weighted gene co-expression network using the R package WGCNA. Cluster analysis indicated the presence of an outlier sample; therefore, subsequent analyses focused on the remaining 266 samples. Our analysis confirmed that the constructed co-expression network adhered to a scale-free topology. Specifically, the logarithm log (*k*) of nodes with connectivity *k* exhibited a negative correlation with the logarithm log [P (*k*)] of the node occurrence probability, achieving a correlation coefficient exceeding 0.8. To ensure the scale-free nature of the network, the optimal soft-thresholding power was determined to be β = 4 (Supplementary Figures S2A, S2B). Subsequently, the expression matrix was transformed into an adjacency matrix, which was then converted into a TOM.

Genes were clustered using the average link hierarchical clustering method. By adhering to the hybrid dynamic tree-cutting standard, a minimum module size of 30 genes was established. After determining the gene modules using the dynamic tree-cutting approach, the eigenvectors for each module were computed. Module clustering was then performed, amalgamating closely related modules into unified modules with a set threshold of height = 0.25. This process resulted in the identification of 13 distinct modules (Figure 3C). The statistics for the number of genes in each module are shown in Supplementary Figure S2C. We assessed the Pearson correlation coefficient between the ME of each module and the phenotypic traits of the samples. The significance of the module increased with increasing correlation coefficients. Supplementary Figure S2D shows the eigengenes of each module with the accompanying list denoting the phenotypic characteristics of each sample. Subsequently, the significance value for each gene module was calculated (Figure 3D). A heightened GS value underscores a module’s increased relevance to cluster1 samples. The “Green” module was the most significant module, and the related genes were displayed in pathway enrichment analysis (Supplementary Figure S2E). From the amalgamated insights derived from the module-phenotypic correlation analyses, the green module emerged as the most pertinent module in relation to m^6^A clusters and immune infiltration. Using the criteria of MM >0.7 and GS > 0.1, 37 hub genes were identified in the green module, which exhibited strong interrelations (Figure 3E).

Subsequently, we evaluated the correlation between the identified 37 hub genes and the proportion of immune-infiltrating cells and m^6^A methylation regulators. Most hub genes demonstrated significant correlations with the proportions of Macrophages M0, as exemplified by *DNMT3B*, *ZNF64*, and *CSTF1* (Figure 3F; Supplementary Figures S2F, S3A). Furthermore, m^6^A regulators were also correlated with the hub genes *DNMT3B*, *ZNF64*, and *CSTF1* (Figure 3G; Supplementary Figures S2G, S3B). Most of the hub genes were significantly correlated with *YTHDF1* and *IGF2BP1*. Notably, *DNMT3B* was positively correlated with *IGF2BP1*, *YTHDF1*, and *VIRMA*, and negatively correlated with *YTHDC2*. Furthermore, we investigated the effects of the hub genes on immune cell infiltration using the TIMER database. Different types of somatic copy number alterations regulate immune cell infiltration into the GC microenvironment. Hub genes and YTHDF1, which are significantly associated with these genes, markedly affected various types of immune-infiltrating cells Figure 3H; Supplementary Figure S2H).

### 3.3 Construction of a prognostic model based on hub genes

**FIGURE 4:**
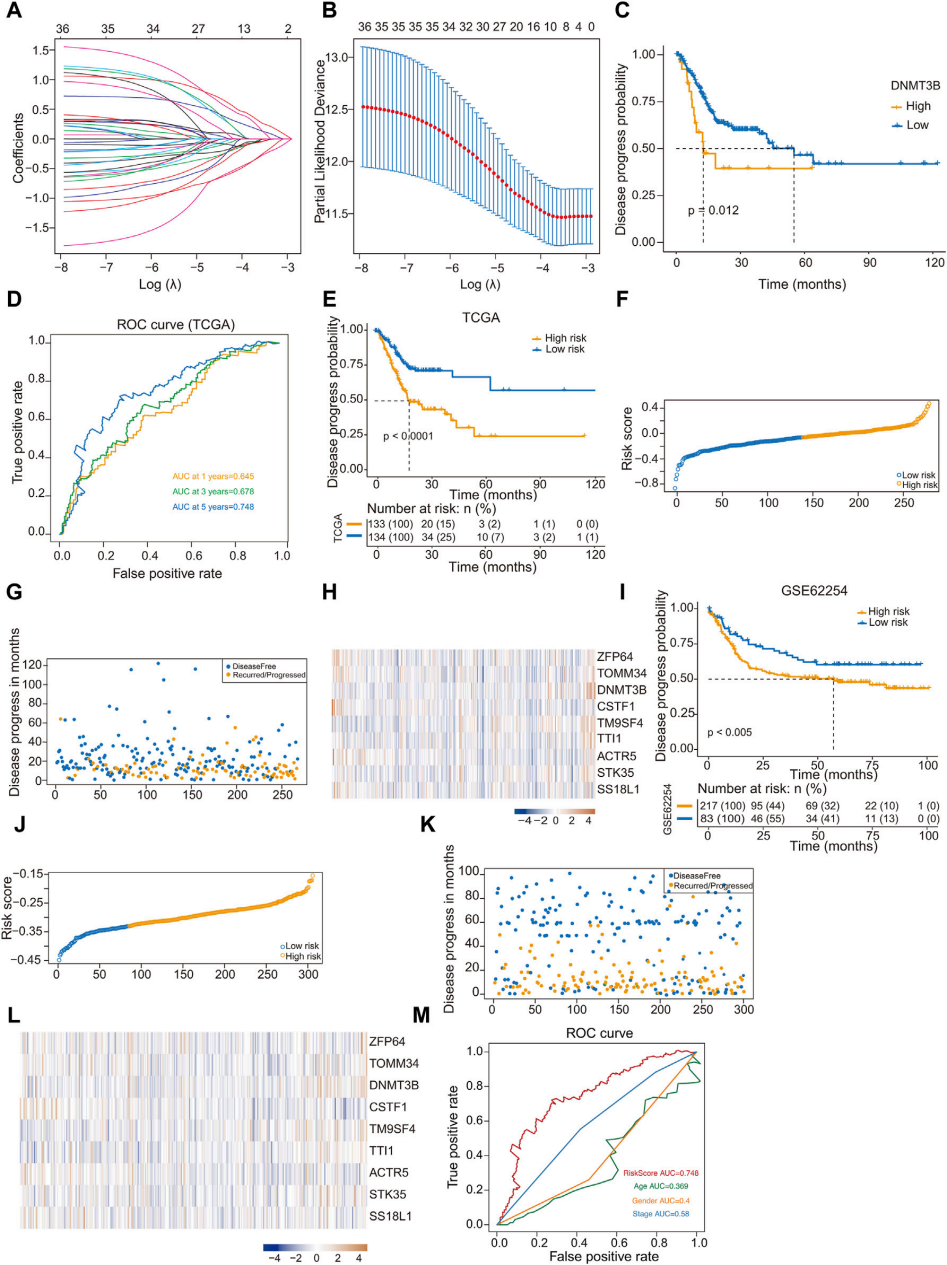
Construction and validation of a prognostic model based on hub genes. **(A)** The trajectory of independent variables in Least Absolute Shrinkage and Selection Operator (LASSO) regression analysis of 9 hub genes in gastric cancer; **(B)** Line plot LASSO regression analysis of 9 hub genes in gastric cancer; **(C)** Kaplan-Meier analysis of DNMT 3B high- and low-expression groups for gastric cancer from TCGA; **(D)** ROC curve of 1-year, 3-year and 5-year survival probability in gastric cancer from TCGA; **(E)** Kaplan-Meier analysis of high-risk and low-risk groups for gastric cancer from TCGA; **(F)** Classify gastric cancer patients into different risk groups according to the median risk score; **(G)** Distribution of risk score in gastric cancer; **(H)** Heatmap of the 9 prognosis-related hub genes expression profiles combined with clinical traits in the high-risk and low-risk groups in the prognostic model; **(I)** Kaplan-Meier analysis of high-risk and low-risk groups for gastric cancer from GSE62254 dataset; **(J)** Classify gastric cancer patients from GSE62254 dataset into different risk groups according to the median risk score; **(K)** Distribution of risk score in gastric cancer; **(L)** Heatmap of the 9 prognosis-related hub genes expression profiles GSE62254 dataset; **(M)** ROC curve of RiskScore, Age, Gender and Stage in gastric cancer from TCGA.

LASSO regression analysis was used to analyze the trajectory of the independent variables, where the *x*-axis represents the logarithm of the variable λ and the *y*-axis denotes the coefficients of the independent variables (Figure 4A). Figure 4B shows the confidence intervals corresponding to each lambda value within the LASSO regression framework. In our subsequent analyses, the survival information of the nine hub genes identified in GC were analyzed, among which the expression of *DNMT3B* is significantly associated with poor prognosis (Figure 4C).

The risk prediction model was assessed using a ROC curve analysis, and the area under the ROC curve values for GC patient samples at 1, 3, and 5 years were 0.645, 0.678, and 0.748, respectively (Figure 4D). We computed the risk score for each sample, and the samples were divided into high- and low-risk group based on the median score. The two groups showed significant difference in prognosis. Kaplan-Meier curve analysis of disease progression probability was conducted to evaluate the effects of the low- and high-risk score groups on prognosis (Figure 4E). Results indicated that the prognosis of high-risk group was worse than that of low-risk one (Figures 4F, G). The heatmap generated from the expression profiles of the nine hub genes in the prognostic model illustrate the expression trends of the differential hub genes as the risk score of the sample increased (Figure 4H).

To validate the efficacy of the risk-scoring model, we conducted a validation using the GSE6254 dataset. Risk scores were computed, based on the optimal cutoff value, samples were categorized into high- and low-risk groups. Similarly, a significant prognostic difference was observed between groups. Kaplan-Meier curve analysis of disease progression probability depicted the low- and high-risk score groups for prognosis in GSE62254 (Figure 4I). The prognosis of high-risk patients was worse than that of low-risk ones (Figures 4J, K). The expression profiles from the GSE62254 dataset of the nine hub genes in the prognostic model are presented in a heatmap (Figure 4L). Compared to Figure 4H, the differential hub genes displayed similar trends as the risk score of the sample increased. Using ROC curves to display different clinical factors and the “RiskScore” to distinguish the survival probability of samples, the results showed that “RiskScore” had the best classification effect (Figure 4M).

### 3.4 Validation of hub genes

**FIGURE 5:**
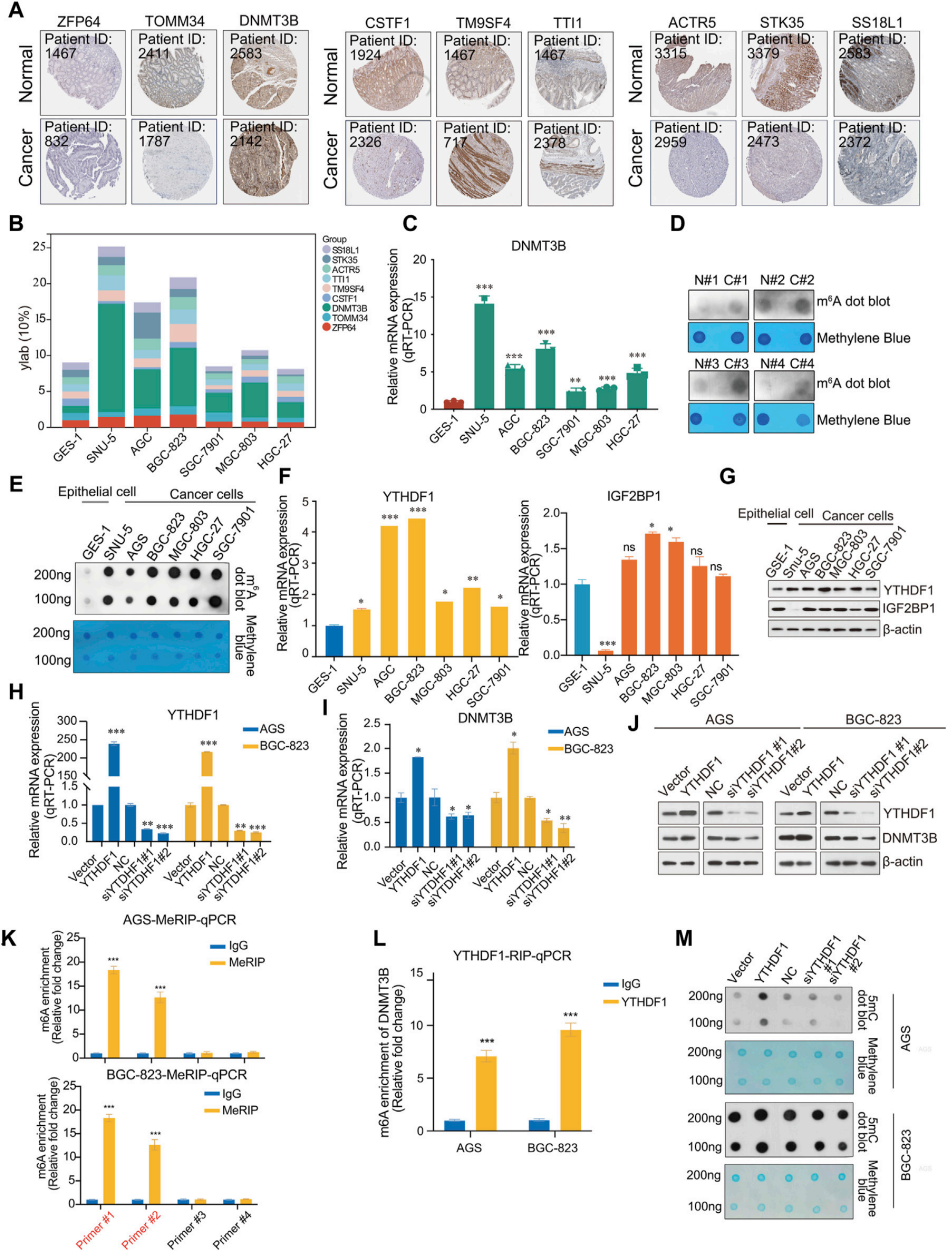
Validation of hub genes. **(A)** Immunohistochemistry (IHC) of 9 hub genes in gastric cancer and normal samples from the HPA database (https://www.proteinatlas.org/); **(B)** Expression profile of 9 hub genes (*ZFP64*, *TOMM34*, *DNMT3B*, *CSTF1*, *TM9SF4*, *TTI1*, *ACTR5*, *STK35*, *SS18L*); **(C)** The mRNA expression of *DNMT3B* in the normal human gastric epithelial cell line GES-1 and gastric cancer cell lines SNU-5, AGS, BGC-823, SGC-7901, MGC-803, HGC-27; **(D)** m^6^A dot blot of total RNA in the normal human gastric epithelial cell line GES-1 and gastric cancer cell lines SNU-5, AGS, BGC-823, SGC-7901, MGC-803, HGC-27; **(E)** m^6^A dot blot of total RNA in the in adjacent and tumor tissues of gastric cancer samples; **(F)** Analysis of the mRNA expression of YTHDF1 and IGF2BP1 in the normal human gastric epithelial cell line GES-1 and gastric cancer cell lines SNU-5, AGS, BGC-823, SGC-7901, MGC-803, HGC-27 via RT-qPCR; **(G)** Analysis of expression of YTHDF1 and IGF2BP1 in the normal human gastric epithelial cell line GES-1 and gastric cancer cell lines SNU-5, AGS, BGC-823, SGC-7901, MGC-803, HGC-27, β-actin served as a loading control; **(H)** Analysis of the proteins from AGS and BGC-823 cells transfected with vector, YTHDF1, or NC, two different siRNA against YTHDF1 were analyzed by Western blot; **(I)** Analysis of the mRNA expression of YTHDF1 in AGS and BGC-823 cells transfected with vector, YTHDF1, or NC, two different siRNA against YTHDF1 were analyzed by RT-qPCR; **(J)** Analysis of the mRNA expression of DNMT3B in AGS and BGC-823 cells transfected with vector, YTHDF1, or NC, two different siRNA against YTHDF1 were analyzed by RT-qPCR; **(K)** Me-RIP assays; **(L)** RIP assays; **(M)** 5mC DNA were analyzed in AGS and BGC-823 cells transfected with vector, YTHDF1, or NC, two different siRNA against YTHDF1 by 5mC dot blot. Error bars represent the mean ± SD of three independent experiments. ∗*p* < 0.05, ∗∗*p* < 0.01, ∗∗∗*p* < 0.001; two-tailed unpaired *t*-test.

To verify the outcome prediction value of these nine hub genes in GC, we obtained their expression patterns in GC samples from the HPA database (https://www.proteinatlas.org/). Consistent with the above results, high expression of DNMT3B, TM9SF4, and TTI1 was observed in GC, whereas the expression of ZFP64, TOMM34, CSTF1, ACTR5, STK35, and SS18L1 was lower in GC (Figure 5A). Among these, DNMT3B, ACTR5 and TM9SF4 demonstrated significant prognostic differences when expressed at high or low levels (Supplementary Figure S4). To further investigate the expression of these nine hub genes in GC, we measured their expression in the normal human gastric epithelial cell GES-1 and in the cancer cell SNU-5, AGS, BGC-823, SGC-7901, MGC-803, and HGC-27 by qPCR. DNMT3B expression was significantly higher in tumor cells than normal epithelial cell GES-1 (Figures 5B, C).

We analyzed the modification levels of m^6^A in normal epithelial and GC cells using m^6^A dot blot experiments. Interestingly, the modification level of m^6^A was higher than that in normal epithelial cells (Figure 5D). Similarly, in RNA extracted from clinical tissue samples, m^6^A modification in tumor tissue was observed to be higher than in adjacent tissues (Figure 5E). Based on these findings, we hypothesize that in the pathogenesis of gastric cancer, there is an upregulation of m^6^A modifications, which in turn regulates key genes critical to the oncogenic processes, thereby facilitating the progression of cancer.

Previous results reported a positive correlation between the expression of *DNMT3B* and that of m^6^A “reader” *YTHDF1* and *IGF2BP1*. We speculate that the modification of m^6^A may lead to an increase in the expression of *DNMT3B*, during which YTHDF1 or IGF2BP1 recognizes the m^6^A modification site of DNMT3B mRNA, thus stabilizing DNMT3B mRNA and promoting the occurrence and development of GC. Therefore, the relationship between m^6^A modification and DNMT3B expression should be further investigated in future research. We detected the expression of YTHDF1 or IGF2BP1 in normal epithelial and tumor cell lines. YTHDF1 was highly expressed in cancer cells, while IGF2BP1 did not exhibit statistically significant differences (Figures 5F, G). To further investigate the molecular mechanism by which YTHDF1 regulates DNMT3B, YTHDF1 was overexpressed and knocked down in AGS and BGC-823 cells. As expected, DNMT3B exhibited changes consistent with those of YTHDF1 (Figures 5H–J). Similarly, we also examined the expression changes of DNMT3B after overexpression or knockdown of IGF2BP1. The results showed that IGF2BP1 did not significantly affect the mRNA expression levels of DNMT3B (Supplementary Figure S5A). Furthermore, by overexpressing or knocking down the m^6^A methyltransferase METTL3 to alter the overall m^6^A modification level in cells, we examined nine hub genes, including DNMT3B. The results showed that an increase in the overall m^6^A modification level upregulated *ZFP64*, *TOMM34*, *DNMT3B*, *TM9SF4*, whereas a decrease downregulated DNMT3B (Supplementary Figure S5B, S5C).

To validate the association between YTHDF1 and DNMT3B mRNA, RIP and MeRIP assays were conducted. Two pairs of primers were designed respectively, according to the two highest confidence m^6^A methylation sites predicted by SRAMP. Subsequently, primer #1 and primer #2 were identified by qPCR (Figure 5K). Then, a direct interaction between YTHDF1 and DNMT3B mRNA was validated by RIP-qPCR with anti-YTHDF1 antibody (Figure 5L). Then we detected the DNA methylation levels in cells overexpressed or knocked down YTHDF1 using 5mC dot blot. We also performed methylene blue staining as a nucleic acid loading control. Accompanied by the overexpression of YTHDF, DNMT3B protein also increases, leading to DNA methylation levels increasing. Similarly, knocking down YTHDF1 also led to a decrease in DNA methylation levels in AGS and BGC-823 cell lines (Figure 5M). Collectively, YTHDF1 promotes DNMT3B protein expression by a direct interaction with DNMT3B mRNA, which resulting a crosstalk between RNA methylation and DNA methylation in GC.

### 3.5 YTHDF1 promotes the progression of GC by regulating DNMT3B

**FIGURE 6:**
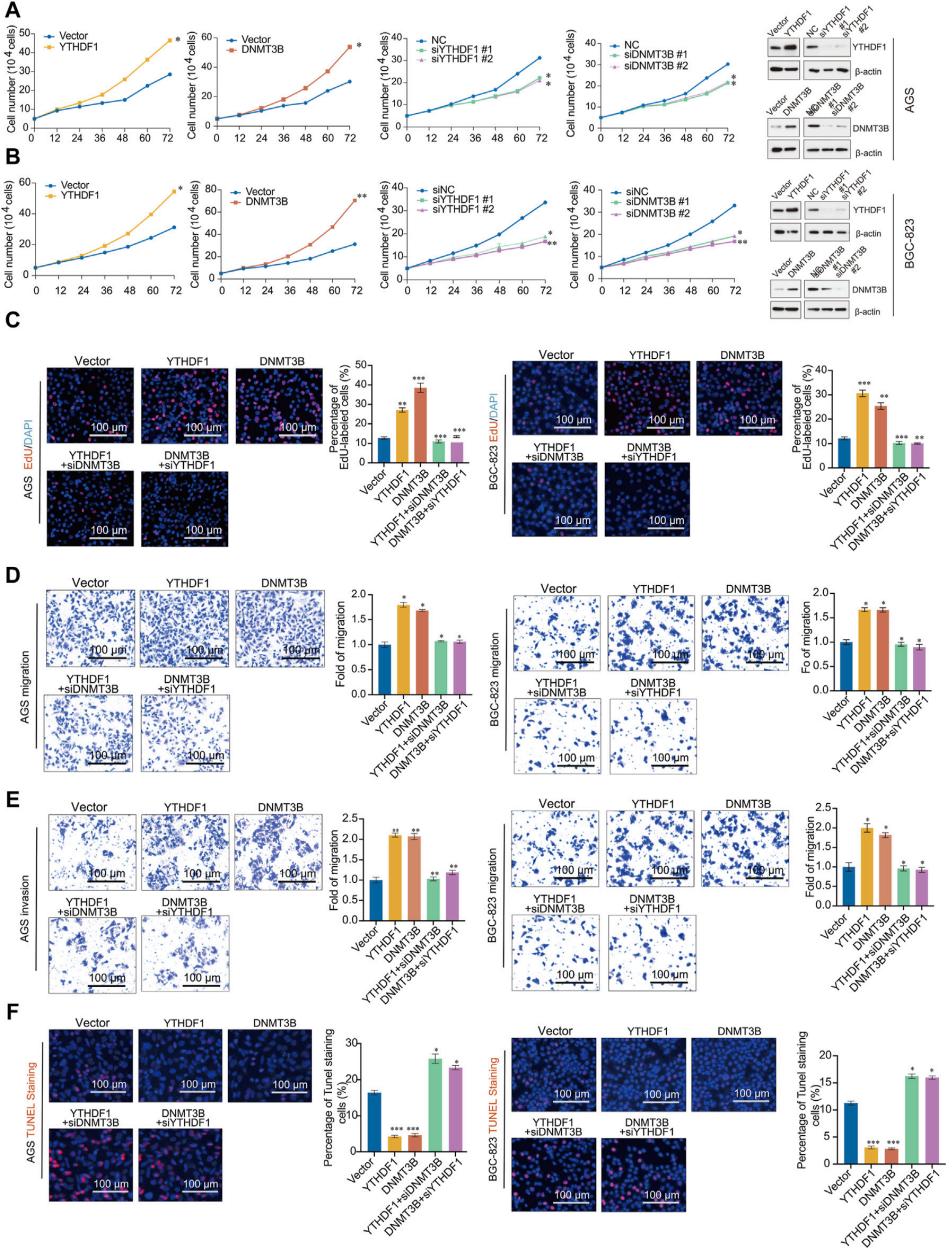
YTHDF1 promotes the occurrence and development of gastric cancer by regulating DNMT3B. **(A,B)** Growth curve analysis was performed in AGS cells transfected with indicated genes; **(C)** EdU incorporation assays were performed in AGS and BGC-823 cells transfected with indicated genes; **(D)** Migration assays assay; **(E)** Invasion assays; **(F)** One step TUNEL apoptosis assays. Error bars represent the mean ± SD of three independent experiments. ∗*p* < 0.05, ∗∗*p* < 0.01, ∗∗∗*p* < 0.001; two-tailed unpaired *t*-test.

Studies have shown that both YTHDF1 and DNMT3B facilitate the tumorigenesis in GC (Wong et al., 2019; Chen et al., 2021; Bai et al., 2022). Consistent with previous reports, the overexpression of YTHDF1 and DNMT3B, respectively, promotes the proliferation of GC cells AGS and BGC-823. Simultaneously, knocking down YTHDF1 results in an inhibition of GC cells (Figures 6A, B). The efficiency of gene overexpression or knockdown is depicted on the right side of the figure. EdU incorporation assays were conducted in AGS and BGC-823 cells transfected with the vector, YTHDF1, DNMT3B, as well as two different siRNAs targeting YTHDF1 or DNMT3B, to assess DNA proliferation. Both YTHDF1 and DNMT3B promoted GC cell proliferation, and the cell proliferation promoted by YTHDF1 overexpression can be reversed by knocking down DNMT3B. On the contrary, knocking down YTHDF1 also attenuated the cell proliferation promotion ability of DNMT3B (Figure 6C).

To further validate the functions of YTHDF1 and DNMT3B in GC, we conducted migration and invasion experiments in AGS and BGC-823 cells. The findings demonstrate that overexpression of YTHDF1 and DNMT3B enhances the migration and invasion of GC cells. Moreover, the migration and invasion abilities promoted by YTHDF1 overexpression can be reversed by knocking down DNMT3B, and knocking down YTHDF1 also reduced the migration and invasion promotion by DNMT3B (Figures 6D, E). To investigate the effects of YTHDF1 and DNMT3B on cell apoptosis, we stained cells transfected with the vector, YTHDF1, DNMT3B, as well as two different siRNAs targeting YTHDF1 or DNMT3B with dUTP labeled with a red fluorescent probe Cyanine 3. The results showed that both YTHDF1 and DNMT3B can inhibit cell apoptosis, while knocking down YTHDF1 or DNMT3B can accelerate cell apoptosis caused by YTHDF1 and DNMT3B (Figure 6F). Thus, YTHDF1 promotes the progression of GC by upregulating DNMT3B, and inhibiting DNMT3B contributes to reduce the tumor promotion ability of YTHDF1.

## 4 Discussion

Cancer progression, driven by genetic and epigenetic aberrations, has received considerable attention. Our exploration of the role of m^6^A modification in GC has further underscored the functions of m^6^A methylation factors, both in the realm of genetic modulation and their interplay with the tumor microenvironment. Controlled by methylation regulators, m^6^A RNA modification patterns influence many key oncological processes, such as tumor proliferation, differentiation, and metastasis, with profound implications (Wang T. et al., 2020; Jiang et al., 2021). Analyzing m^6^A modification patterns in GC samples revealed distinct subgroups for diagnosis, guiding therapeutic strategies and ensuring personalized patient care.

Our findings highlight the critical role of immune cell infiltration in GC prognosis. The differences in prognosis between the high- and low-infiltration cohorts highlight the crucial influence of the TME on disease outcomes. This is congruent with current oncology paradigms that emphasize the significance of immune-tumor interactions. The identification of the “green” module via WGCNA showcases a direct association between the m^6^A RNA methylation cluster and immune infiltration patterns. This intersection of epigenetic regulation and immune dynamics underscores the holistic nature of cancer progression.

Establishing a risk prediction model based on a selected group of hub genes has potential clinical utility. Upon validation in larger and more diverse patient cohorts, this model could emerge as a reliable tool for risk stratification in patients with GC. The nine hub genes used to establish the risk prediction model are vital for cancer progression. *ZFP64* enhances the activation of the p65 subunit, thereby promoting the production of pro-inflammatory and type-I interferons by Toll-like receptor-activated macrophages (Wang et al., 2013). Overexpression of *ZFP64* promotes the proliferation of lung adenocarcinoma cells through activating the Notch pathway and is associated with poor prognosis (Jiang et al., 2020). ZFP64 functions as a transcription factor that promotes the expression of Galectin-1 (GAL-1), contributing to stem-cell-like properties and an immunosuppressive tumor environment. This activity enhances resistance to the chemotherapy drug nab-paclitaxel, playing a key role in the progression and chemoresistance of gastric cancer (Zhu et al., 2022). CD4^+^ T cells specifically induce the expression of mitochondrial TOMM34 (Gerner et al., 2019), and the role of *TOMM34* in cancer cell growth suggests its potential in anti-cancer drug development or colorectal cancer diagnosis (Shimokawa et al., 2006). TOMM34 was identified as differentially expressed between intestinal-type and diffuse-type gastric cancer, suggesting it plays a role in the distinct molecular pathways of these cancer subtypes. This involvement may relate to processes important in cancer progression, such as adaptation to stress and resistance to therapy (Tanabe et al., 2020). *DNMT3B* is widely overexpressed in non-small cell lung cancer (NSCLC) and may be a potential molecular biomarker for personalized therapy (Samakoglu et al., 2012). DNMT3B influences tumor development through its enzymatic activity. Specifically, S-nitrosylation of DNMT3B reduces its enzymatic activity, leading to an abnormal upregulation of the Cyclin D2 gene (*CCND2*), which is necessary for the proliferation of certain tumor cells (Okuda et al., 2023). In gastric cancer, DNMT3B promotes tumor progression by methylating the *MYH11* gene, thereby decreasing its expression and allowing the increase of TNFRSF14, which supports cancer development. This highlights DNMT3B as a potential target for cancer therapy (Wang et al., 2021). *CSTF1*, pivotal in DNA damage repair, is linked to increased breast cancer risk in BRCA2 mutation carriers due to *CSTF1* mutations (Paolillo et al., 2015). In the study on gastrointestinal stromal tumors (GISTs), CSTF1 was involved in a fusion with Aurora kinase A (AURKA). This suggests that CSTF1, through this fusion, could play a role in the progression or behavior of GISTs, although specific mechanisms were not detailed (Denu et al., 2024). TM9SF4, primarily involved in cell adhesion and innate immunity, is overexpressed in a small subset of patients with metastatic melanoma, acute myeloid leukemia, and myelodysplastic syndromes (Paolillo et al., 2015). TM9SF4 was identified as a key gene in the regulatory network affecting response to cisplatin and fluorouracil treatment. Its specific role isn’t detailed, but its prominence in the network suggests it may influence mechanisms underlying chemoresistance (Sun et al., 2021). The interaction of Tti1 with mTOR in both mTORC1 and mTORC2 complexes regulates autophagy suppression (Kaizuka et al., 2010). ACTR5 has a pro-tumorigenic effect in neuroblastoma, and the knockdown of *ACTR5* reduces cell proliferation and differentiation abilities (Veschi et al., 2017). STK35 regulates apoptosis and proliferation in osteosarcoma cells in osteosarcoma, exhibiting oncogenic properties (Wu et al., 2018). STK35 has been linked to immune signatures in gastric cancer, suggesting it may impact the immune response and effectiveness of immunotherapy. This connection highlights STK35 as a potential target for improving treatment outcomes (He and Wang, 2020). *SS18L1* is associated with the occurrence and development of endometrial serous carcinoma (Saglam et al., 2020). SS18L1 has been identified as having copy number variations significantly linked to tumor metastasis. This association suggests that SS18L1 may influence the spread of gastric cancer, making it a potential marker or target for therapeutic strategies (Zhu et al., 2020). While these genes are implicated in tumor development and progression, their specific mechanisms in GC remain incompletely understood. Therefore, the nine hub genes identified through integration of m^6^A modification characteristics and the TME may play important roles in the prognostic assessment of patients with GC.

Furthermore, we found that DNMT3B was positively correlated with IGF2BP1 and YTHDF1, upon evaluating the correlation of these 37 hub genes with m^6^A methylation regulators and the proportion of immune-infiltrating cells. In a series of molecular experiments and cellular phenotypic validations, we demonstrated that DNMT3B and YTHDF1 cooperate to promote the proliferation, invasion, and metastasis of GC cells. YTHDF1 plays an important role in GC progression, and its functions and molecular mechanisms have been extensively investigated. High expression of YTHDF1 is associated with more aggressive tumor progression and poor prognosis in GC. Engineered small extracellular vesicles targeting YTHDF1 efficiently suppress GC progression and metastasis through epigenetic and immune modulation (You et al., 2023). The loss of YTHDF1 in gastric tumors potentiates the antitumor immune response by promoting the infiltration of mature dendritic cells (Bai et al., 2022). Elevated YTHDF1 expression also acts as a shield against the antitumor effects of chemotherapy and immunotherapy (Chen et al., 2022). Moreover, YTHDF1 overexpression holds clinical diagnostic significance across various cancers, including NSCLC, breast cancer, cervical cancer, GC, and colorectal cancer (Zhu Y. et al., 2023). YTHDF1 is significantly associated with metastatic gene signatures through ARHGEF2 translation and RhoA signaling activation in colorectal cancer (Wang et al., 2022). YTHDF1 directly targets p65 mRNA, promoting p65 protein overexpression without altering mRNA levels in Ythdf1-KO cells (Bao et al., 2023). YTHDF1 promotes cancer stem cell renewal and resistance to tyrosine kinase inhibitors in hepatocellular carcinoma (HCC), which enhances the stability and translation of m^6^A-modified NOTCH1 mRNA, leading to increased expression of NOTCH1 target genes. YTHDF1 drives HCC stemness and drug resistance, making it a potential therapeutic target for HCC treatment (Zhang et al., 2024). YTHDF1 promotes migration, invasion, and osteoblast adhesion and induces osteoclast differentiation of cancer cells *in vitro* and *in vivo* by inducing EZH2 and CDH11 translation (Wang et al., 2024).

In conclusion, our study highlights the intricate ties between m^6^A RNA methylation and TME dynamics in GC. As we move toward precision medicine, such insights will be pivotal in driving therapeutic innovations and improving patient outcomes. In addition, utilizing MeRIP and RIP experiments, we elucidated the molecular mechanism underlying the regulation of DNMT3B expression by m^6^A “reader”.

YTHDF1 and explored the crosstalk between m^6^A modification and 5mC modification in GC cells. Although our findings are promising, further investigations are essential to fully understand the mechanistic underpinnings and translate these insights into applicable clinical strategies.

## Data Availability

The datasets presented in this study can be found in online repositories. The names of the repository/repositories and accession number(s) can be found in the article/Supplementary Material.

## Abbreviations

DMEM, Dulbecco’s modified Eagle’s medium; GC, Gastric cancer; GS, Gene significance; ME, Module eigengene; MM, Module membership; NC, Negative control; NSCLC, Non-small cell lung cancer; PCA, Principal Component Analysis; TME, Tumor microenvironment; TCGA, The Cancer Genome Atlas-Stomach Adenocarcinoma; TOM, Topological Overlap Measure; WGCNA, Weighted Gene Co-expression Network Analysis.
