# Supplementary material for: Prognostic analysis of patients with gastric cancer based on N6-methyladenosine modification patterns and tumor microenvironment characterization
Source: Front Pharmacol. 2024 Aug 9;15:1445321. doi: 10.3389/fphar.2024.1445321 (PMC11341457; doi:10.3389/fphar.2024.1445321)
Supplement: Supplementary file 1 [file Table1.DOCX]

Supplementary Material

**Supplementary Tables**

Table S1. The list of the 31 m^6^A regulators.

| Category | Genes |
| --- | --- |
| “Writers” | METTL3, METTL14, METTL16, WTAP, VIRMA(KIAA1429), RBMX, ZC3H13, RBM15, RBM15B, ZCCHC4, CBLL1 |
| “Readers” | YTHDC1, YTHDC2, YTHDF1, YTHDF2, YTHDF3, HNRNPC, HNRNPA2B1, IGF2BP1, IGF2BP2, IGF2BP3, EIF3A, ELAVL1, LRPPRC, PRRC2A, SND1, FMR1 |
| “Erasers” | FTO, ALKBH5 |
| Others | G3BP1, G3BP2 |

Table S2. The source and culture condition of cell lines

| Cell Line | Culture Medium | Source | RRID |
| --- | --- | --- | --- |
| GES-1 | DMEM/F12+10% FBS | Cell Bank Chinese Academy of Sciences | CVCL_EQ2 |
| SNU-5 | DMEM/F12+10% FBS | Cell Bank Chinese Academy of Sciences | CVCL_0078 |
| AGS | DMEM/F12+10% FBS | Shanghai Institute of Biochemical Cell Science | CVCL_0139 |
| BGC-823 | DMEM+10% FBS | Shanghai Institute of Biochemical Cell Science | CVCL_3360 |
| MGC-803 | DMEM+10% FBS | Cell Bank Chinese Academy of Sciences | CVCL_5334 |
| HGC-27 | DMEM+10% FBS | Shanghai Institute of Biochemical Cell Science | CVCL_1279 |
| SGC-7901 | DMEM+10% FBS | Cell Bank Chinese Academy of Sciences | CVCL_0520 |

Table S3. The siRNA sequences.

| siRNA | 5' to 3' sequence | 5' to 3' sequence |
| --- | --- | --- |
| siYTHDF1#1 | CCCGAAAGAGUUUGAGUGGTT | CCACUCAAACUCUUUCGGGTT |
| siYTHDF1#2 | GUUCGUUACAUCAGAAGGATT | UCCUUCUGAUGUAACGAACTT |
| siYTHDF1#3 | CGGUGGGACAAAUGUGAACTT | GUUCACAUUUGUCCCACCGTT |
| siDNMT3B#1 | CCAAGCGCCUCAAGACAAATT | UUUGUCUUGAGGCGCUUGGTT |
| siDNMT3B#2 | CCCAUUCGAGUCCUGUCAUTT | AUGACAGGACUCGAAUGGGTT |
| siDNMT3B#3 | CCAUGAAGGUUGGCGACAATT | UUGUCGCCAACCUUCAUGGTT |

Table S4. The primers used in qPCR.

| Gene | Forward primer (5' to 3') | Reverse primer (5' to 3') |
| --- | --- | --- |
| *YTHDF1* | CGTGGACACCCAGAGAACAA | TAGCTGGACAGGTAGGGGTC |
| *ZFP64* | CATGTAGATCAGCGTCCCCC | TTGTGCCACCTGGAATTTGC |
| *TOMM34* | GATGGCCCCCAAATTCCCA | GGGTCTGAAGAACCTTGCG |
| *DNMT3B* | CCGCTTCCTCGCAGCAG | TCCCTTCATGCTTTCCTGCC |
| *CSTF1* | TGAAGCAACAGCCCCTTGAC | TGTCATCGTTTTCCATTCCGAG |
| *TM9SF4* | TGAGTGAAGGAGACTCCGGG | ACCACGGCAACCAATCAGTG |
| *TTI1* | GGCTGGAAGACGAGCCTG | AGCTGAACACAGACTGGACG |
| *ACTR5* | GCCCGTGTTTAACTTGGCAG | TGTCCTTTGGGTACCTGTCC |
| *STK35* | TCAAGAAGATCCGCTGCGAC | AACCCAGGATCCTTTCTCCTTTC |
| *SS18L1* | CAAGAGGCAAAGGGGAGGTT | CAGGATCTGCTGGTACTGCG |
| *β-actin* | AAGATCAAGATCATTGCTCCTC | CATAGTCCGCCTAGAAGCA |

Table S5. The primers of DNMT3B used in MeRIP-qPCR.

| Primer | Forward primer (5' to 3') | Reverse primer (5' to 3') |
| --- | --- | --- |
| Primer #1 | TCTCCAAGAGGGAGGTGTCC | CAGAGCCATCCCCATCTTCC |
| Primer #2 | CGACTCTCCAAGAGGGAGGT | GTGTCAGAGCCATCCCCATC |
| Primer #3 | TTACATGTGTCTCCCGCAGC | CCCGTGTCACTGGTGAAGAA |
| Primer #4 | TGTTACATGTGTCTCCCGCA | GTGTCACTGGTGAAGAAGGC |
