# Supplementary material for: Prognostic analysis of patients with gastric cancer based on N6-methyladenosine modification patterns and tumor microenvironment characterization
Source: Front Pharmacol. 2024 Aug 9;15:1445321. doi: 10.3389/fphar.2024.1445321 (PMC11341457; doi:10.3389/fphar.2024.1445321)
Supplement: Supplementary file 2 [file DataSheet1.PDF]

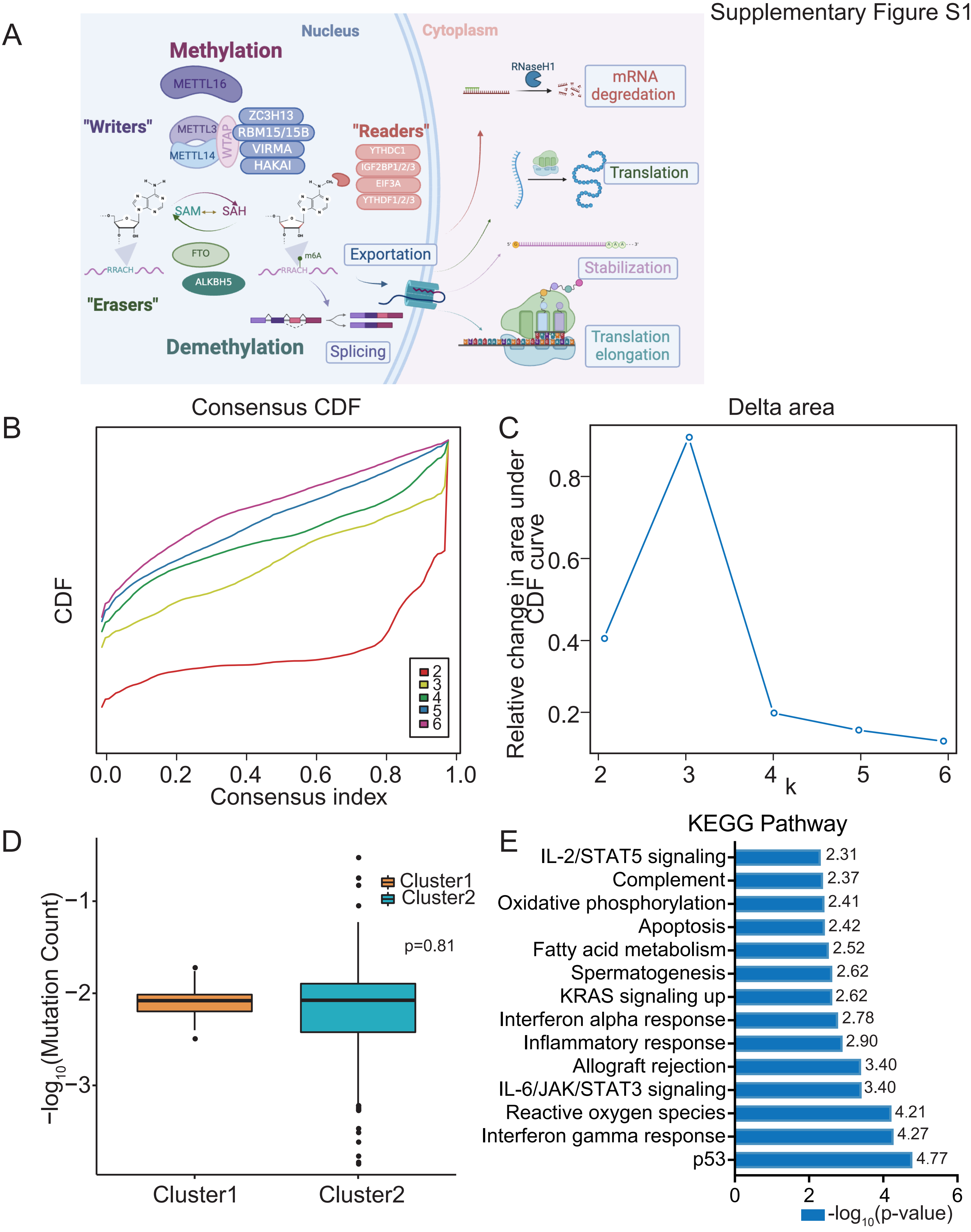
A
Nucleus
Cytoplasm
Methylation
METTL16
"Writers"
METTL3
METTL14
WTAP
ZC3H13
RBM15/15B
VIRMA
HAKAI
"Readers"
YTHDC1
IGF2BP1/2/3
EIF3A
YTHDF1/2/3
SAM SAH
FTO
ALKBH5
RRACH
m6A
"Erasers"
Demethylation
Exportation
Splicing
RNaseH1
mRNA degredation
Translation
Stabilization
Translation elongation
B
Consensus CDF
CDF
Consensus index
0.0 0.2 0.4 0.6 0.8 1.0
2 3 4 5 6
C
Delta area
Relative change in area under CDF curve
0.2 0.4 0.6 0.8
k
2 3 4 5 6
D
−log10(Mutation Count)
−1 −2 −3
Cluster1
Cluster2
p=0.81
E
KEGG Pathway
IL-2/STAT5 signaling 2.31
Complement 2.37
Oxidative phosphorylation 2.41
Apoptosis 2.42
Fatty acid metabolism 2.52
Spermatogenesis 2.62
KRAS signaling up 2.62
Interferon alpha response 2.78
Inflammatory response 2.90
Allograft rejection 3.40
IL-6/JAK/STAT3 signaling 3.40
Reactive oxygen species 4.21
Interferon gamma response 4.27
p53 4.77
0 2 4 6
-log10(p-value)

Supplementary Figure S2

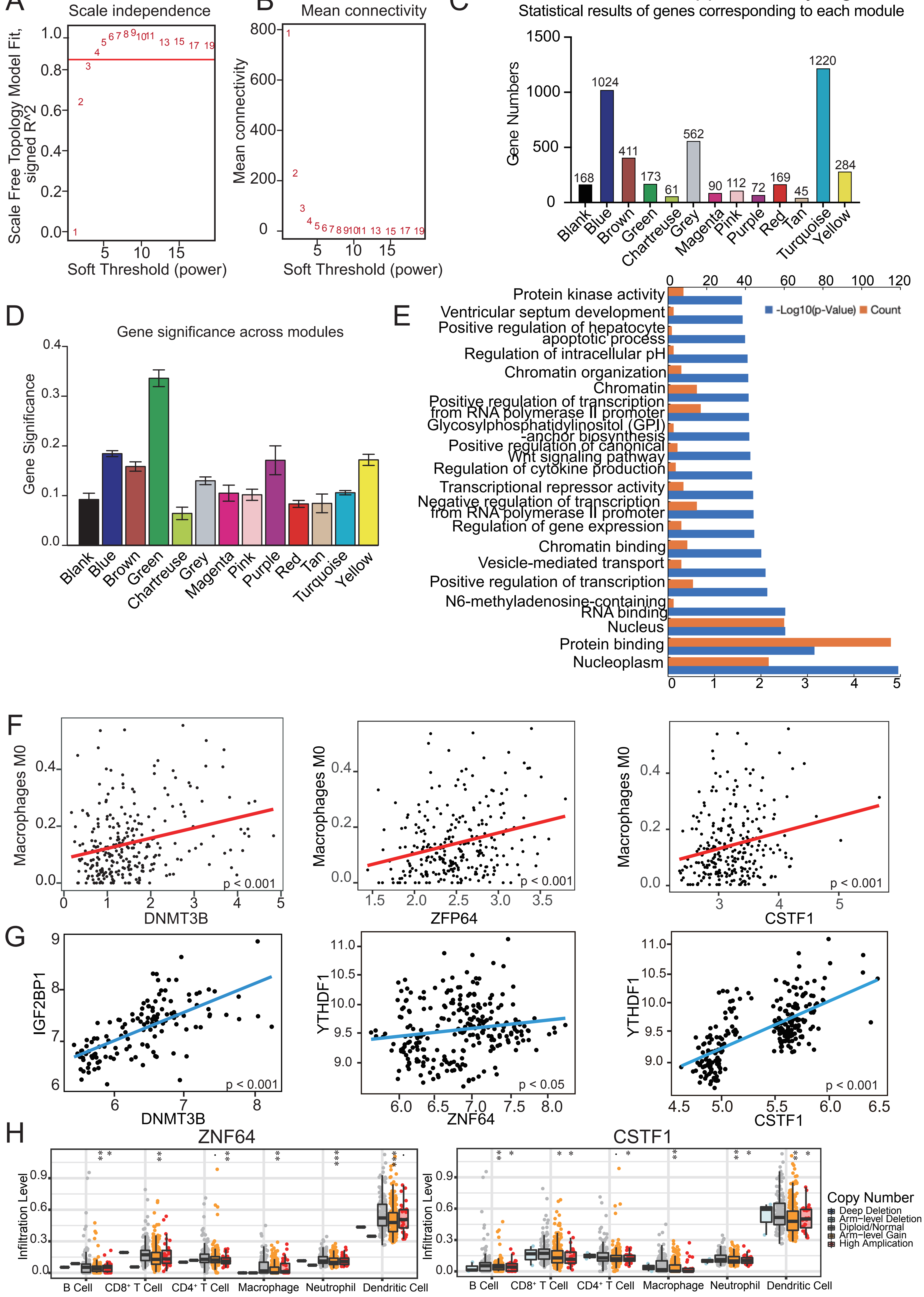

Supplementary Figure S3

A

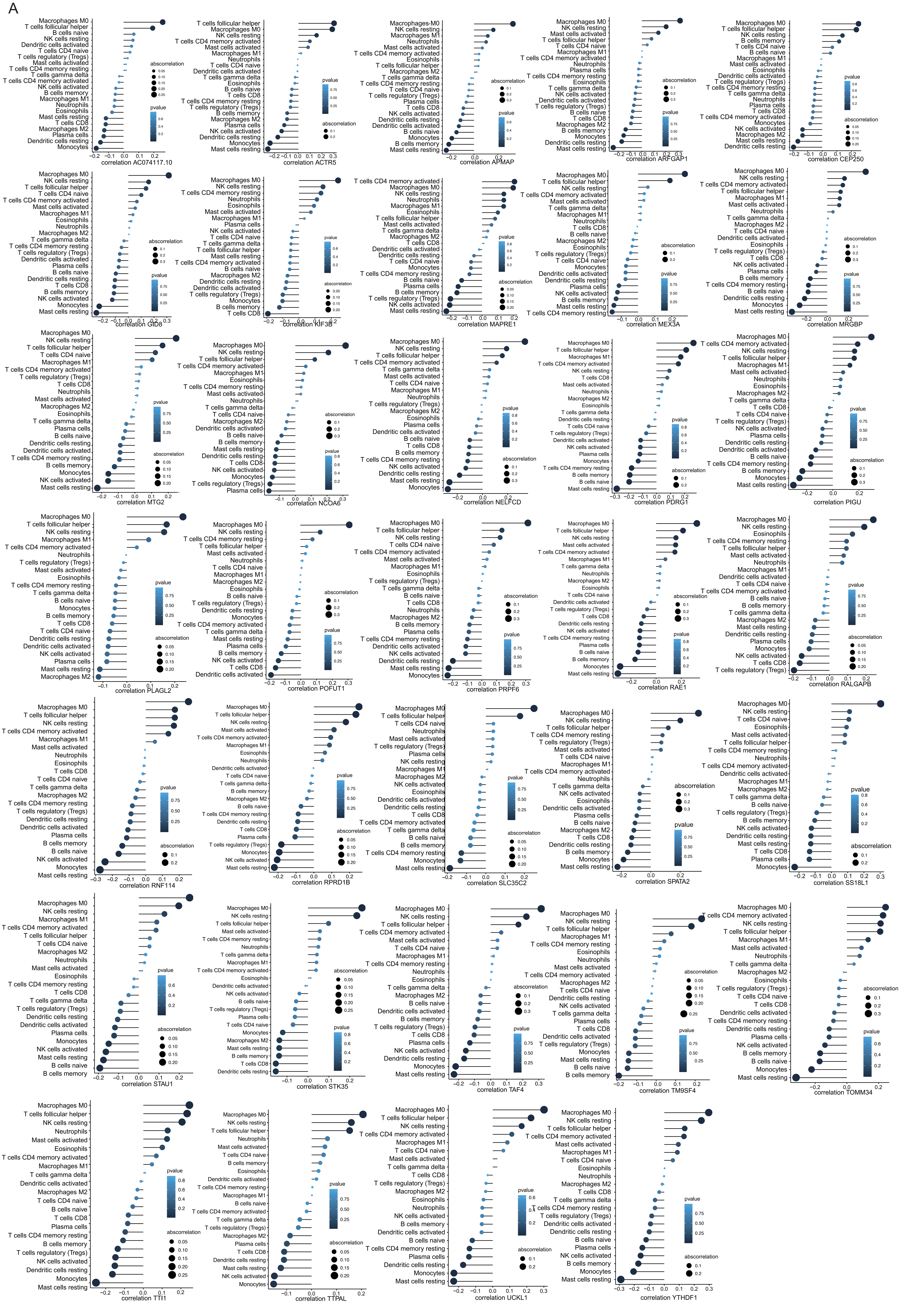

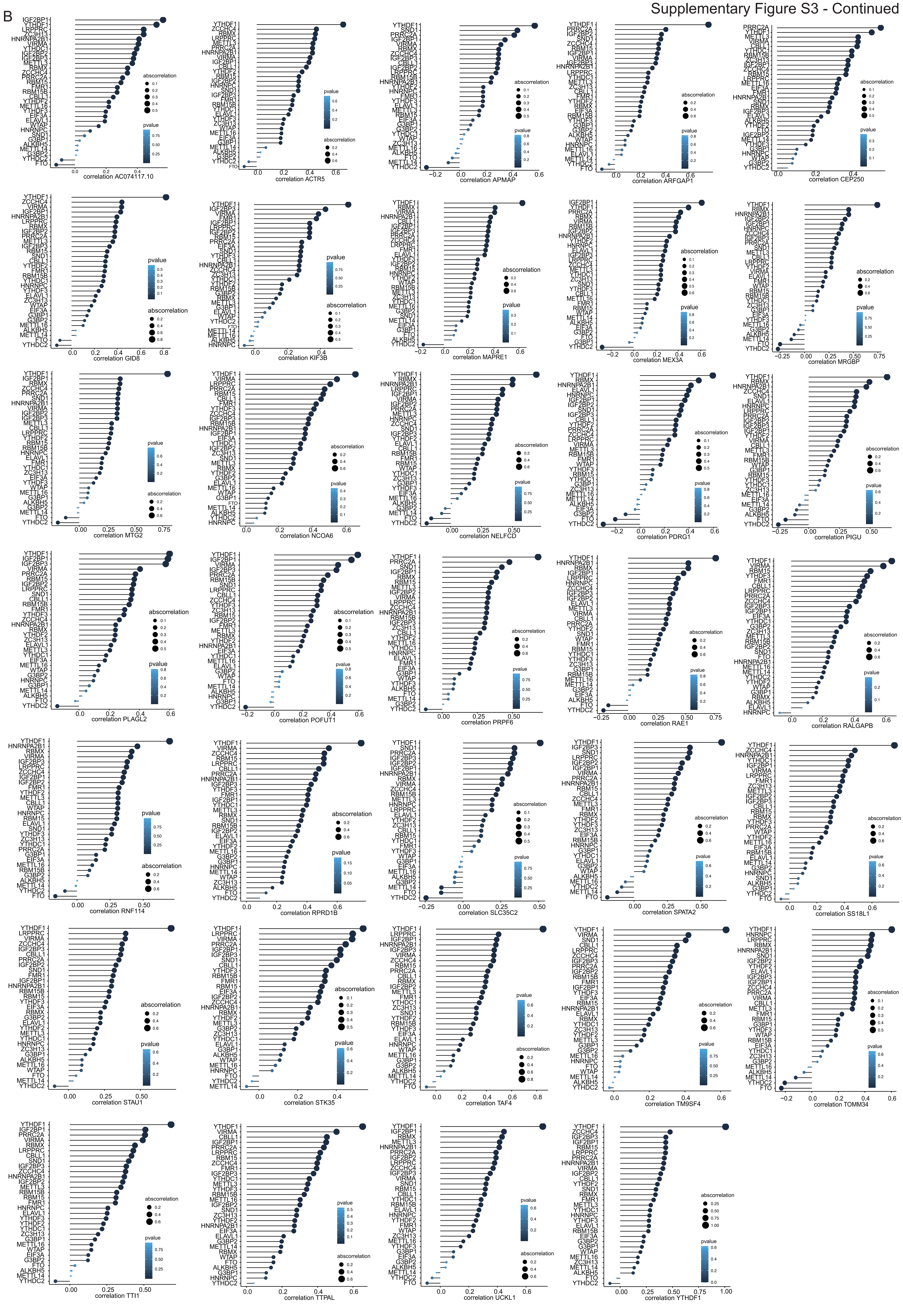
B
correlation AC074117.10
correlation ACTR5
correlation APMAP
correlation ARFGAP1
correlation CEP250
correlation GID8
correlation KIF3B
correlation MAPRE1
correlation MEX3A
correlation MRGBP
correlation MTG2
correlation NCOA6
correlation NELFCD
correlation PDRG1
correlation PIGU
correlation PLAGL2
correlation POFUT1
correlation PRPF6
correlation RAE1
correlation RALGAPB
correlation RNF114
correlation RPRD1B
correlation SLC35C2
correlation SPATA2
correlation SS18L1
correlation STAU1
correlation STK35
correlation TAF4
correlation TM9SF4
correlation TOMM34
correlation TTI1
correlation TTPAL
correlation UCKL1
correlation YTHDF1
abscorrelation
pvalue

Supplementary Figure S4

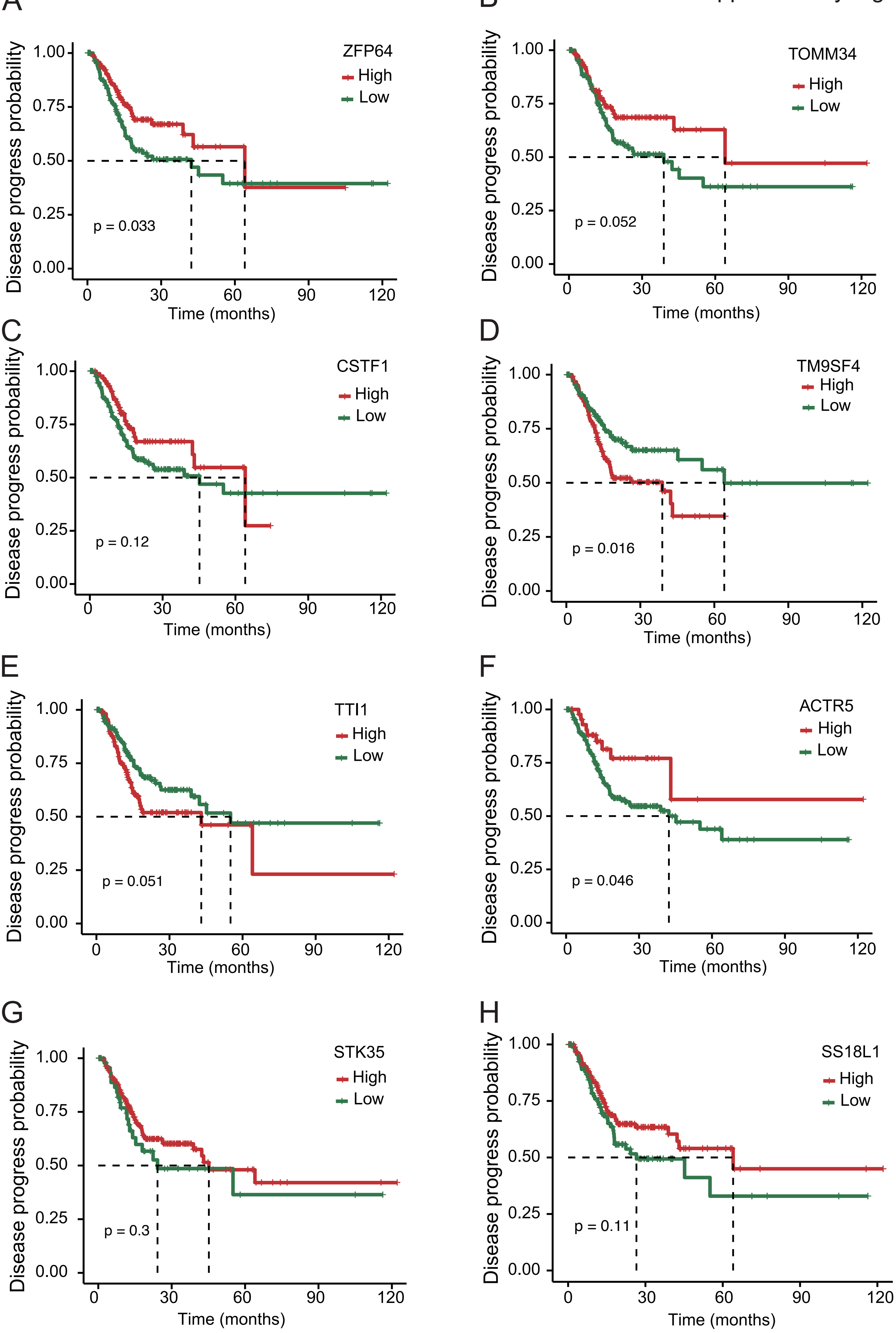

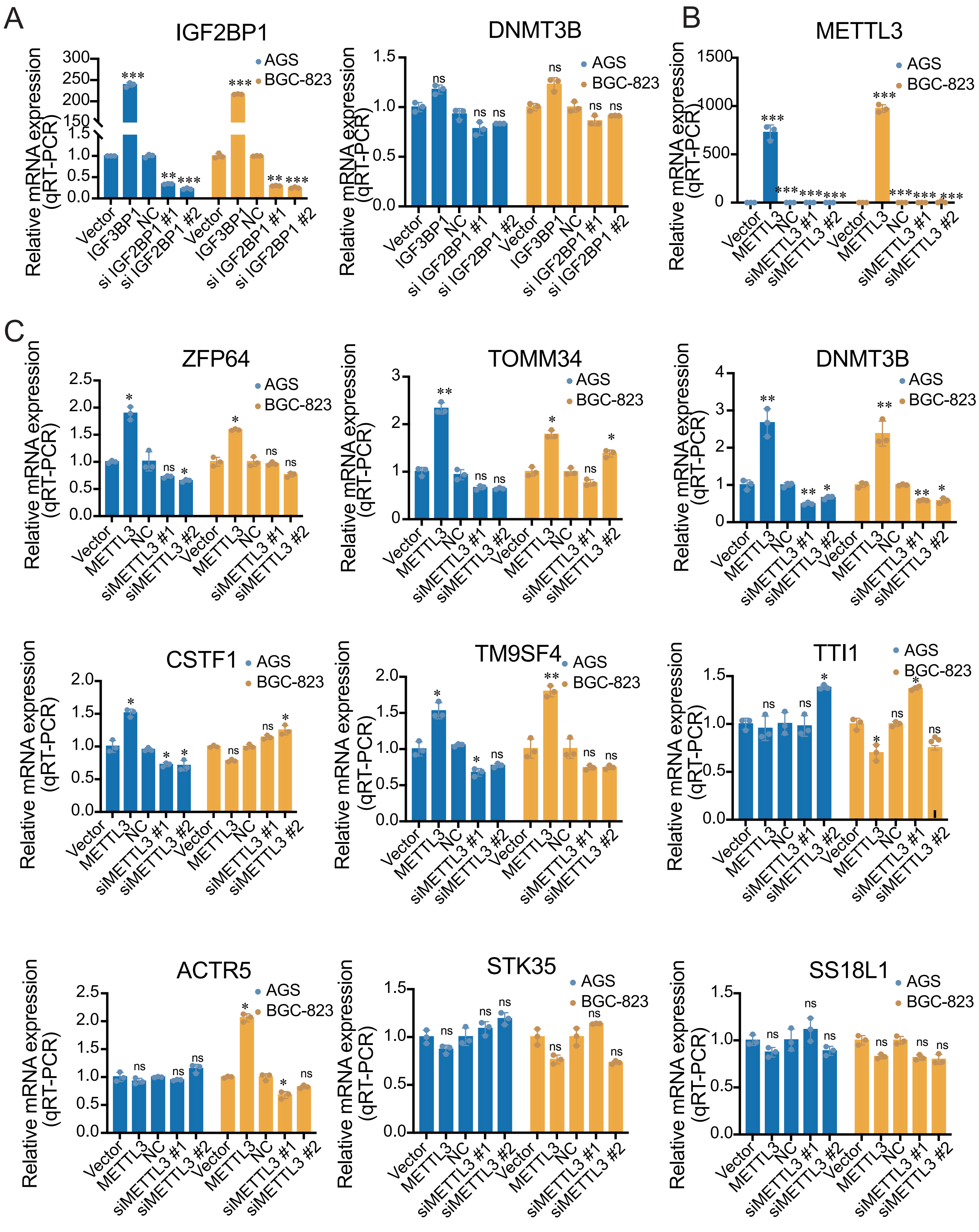
A
IGF2BP1
DNMT3B
B
METTL3
C
ZFP64
TOMM34
DNMT3B
CSTF1
TM9SF4
TTI1
ACTR5
STK35
SS18L1
AGS
BGC-823
Relative mRNA expression (qRT-PCR)
Vector
IGF3BP1
NC
si IGF2BP1 #1
si IGF2BP1 #2
METTL3
siMETTL3 #1
siMETTL3 #2
ns

Supplementary Figure S1. The crosstalk of m^{6}A methylation modification patterns and Landscape of TME in GC.

(A) Summary of the dynamic reversible process of m^{6}A methylation mediated by regulators； (B) Clustering cumulative distribution function; (C) Delta Area Plot; (D) No significant difference in the number of mutations between the two groups. (E) Pathway enrichment analysis of distinct subgroups. The asterisks represented the statistical p value (*p < 0.05; **p < 0.01; ***p < 0.001)

Supplementary Figure S2. Correlation of hub genes with m^{6}A regulators and immune infiltration.

(A,B)Analysis of network topology for various soft-thresholding powers; (C) The genes number of each module; (D) Bar plot of mean significance across modules; (E) Pathway enrichment analysis of the genes of "Green" module; (F)The correlation between macrophages M0 and hub genes DNMT3B, ZNF64, CSTF1; (G) The correlation between m^{6}A regulators YTHDF1 and hub genes DNMT3B, ZNF64, CSTF1;(H) Analysis of the differences in immune infiltration and SCNAs among hub genes. ∗p <0.05, ∗∗p < 0.01, ∗∗∗p < 0.001; two-tailed unpaired *t* test.

Supplementary Figure S3. The correlations analysis of hub genes.

(A) The correlations between other hub genes and the proportions of immune infiltrating cells; (B) The correlations between other hub genes and 31 m^{6}A RNA methylation regulators.

Supplementary Figure S4. The mRNA expression profile of hub genes.

(A) Analysis of the mRNA expression of IGF2BP1 and DNMT3B in AGS and BGC-823 cells transfected with vector, IGF2BP1, or NC, two different siRNA against IGF2BP1 were analyzed by RT-qPCR; (B) Analysis of the mRNA expression of METTL3 in AGS and BGC-823 cells transfected with vector, METTL3, or NC, two different siRNA against METTL3 were analyzed by RT-qPCR; (C) Analysis of the mRNA expression of 9 hub genes in AGS and BGC-823 cells transfected with vector, METTL3, or NC, two different siRNA against METTL3 were analyzed by RT-qPCR.

Supplementary Figure S5. Kaplan-Meier analysis of other hub genes high- and low-expression groups for gastric cancer from TCGA.
